# A spleen tyrosine kinase inhibitor attenuates the proliferation and migration of vascular smooth muscle cells

**DOI:** 10.1186/s40659-016-0106-3

**Published:** 2017-01-18

**Authors:** Hyang-Hee Seo, Sang Woo Kim, Chang Youn Lee, Kyu Hee Lim, Jiyun Lee, Eunhyun Choi, Soyeon Lim, Seahyoung Lee, Ki-Chul Hwang

**Affiliations:** 1 Brain Korea 21 PLUS Project for Medical Science, Yonsei University, Seoul, South Korea; 2 Institute for Bio-Medical Convergence, College of Medicine, Catholic Kwandong University, Gangneung-si, Gangwon-do South Korea; 3 Department of Integrated Omics for Biomedical Sciences, Yonsei University, Seoul, South Korea; 4 Department of Veterinary Physiology, College of Veterinary Medicine, Chonbuk National University, Jeonju, Jeollabuk-Do South Korea

**Keywords:** Syk kinase inhibitor, BAY61-3606, VSMC, Proliferation, Migration

## Abstract

**Background:**

Pathologic vascular smooth muscle cell (VSMC) proliferation and migration after vascular injury promotes the development of occlusive vascular disease. Therefore, an effective chemical agent to suppress aberrant proliferation and migration of VSMCs can be a potential therapeutic modality for occlusive vascular disease such as atherosclerosis and restenosis. To find an anti-proliferative chemical agent for VSMCs, we screened an in-house small molecule library, and the selected small molecule was further validated for its anti-proliferative effect on VSMCs using multiple approaches, such as cell proliferation assays, wound healing assays, transwell migration assays, and ex vivo aortic ring assay.

**Results:**

Among 43 initially screened small molecule inhibitors of kinases that have no known anti-proliferative effect on VSMCs, a spleen tyrosine kinase (Syk) inhibitor (BAY61-3606) showed significant anti-proliferative effect on VSMCs. Further experiments indicated that BAY61 attenuated the VSMC proliferation in both concentration- and time-dependent manner, and it also significantly suppressed the migration of VSMCs as assessed by both wound healing assays and transwell assays. Additionally, BAY61 suppressed the sprouting of VSMCs from endothelium-removed aortic rings.

**Conclusion:**

The present study identified a Syk kinase inhibitor as a potent VSMC proliferation and migration inhibitor and warrants further studies to elucidate its underlying molecular mechanisms, such as its primary target, and to validate its in vivo efficacy as a therapeutic agent for restenosis and atherosclerosis.

## Background

Normal vascular smooth muscle cells (VSMCs) maintain a minimal proliferation rate and active contractility [1]. However, non-physiologic stimuli, such as excessive cytokines, shear stress, vascular injury, or inflammation, can promote the phenotypic switch of VSMCs from a contractile type to a synthetic phenotype [2, 3], leading to the development of occlusive vascular diseases [4, 5]. Such aberrant proliferation and migration of VSMCs limit the long-term efficacy of the clinical interventions such as angioplasty, stents, and coronary bypass [6]. To overcome this problem of excessive VSMC proliferation, advanced approaches (i.e., drug-eluting stents) have been tried and produced some promising results [7]. Nevertheless, the incidence of drug-eluding stent failure still remains at approximately 10%, [7, 8]. Furthermore, although different modalities, including calcium channel blockers, antiplatelet drugs, and angiotensin converting enzyme inhibitors, have been tried to control occlusive vascular disease, the results were not consistent [9]. Therefore, searching alternative means to control the aberrant proliferation and migration of VSMCs is still an important task for developing an optimized therapeutics to treat occlusive disease.

In the present study, we screened number of small molecule inhibitors of six major kinase subfamilies, to find an alternative therapeutic agent for suppressing VSMC proliferation and migration based on the results of multiple experiments, such as cell proliferation assays, wound healing assays, and transwell migration assays, that assessed the anti-proliferative and migratory effect on in vitro. The effect of the selected small molecule on the sprouting of VSMCs from endothelium-denuded aortic rings was also examined. The results indicated that the selected small molecule, a spleen tyrosine kinase (Syk) inhibitor (BAY61-3606), is an effective agent for suppressing proliferation and migration of VSMCs in vitro and ex vivo and that has therapeutic potential for treating occlusive vascular disease involving aberrant proliferation and migration of VSMCs.

## Results

### Initial screening of small molecules for anti-proliferative effect on VSMCs

**Fig. 1:**
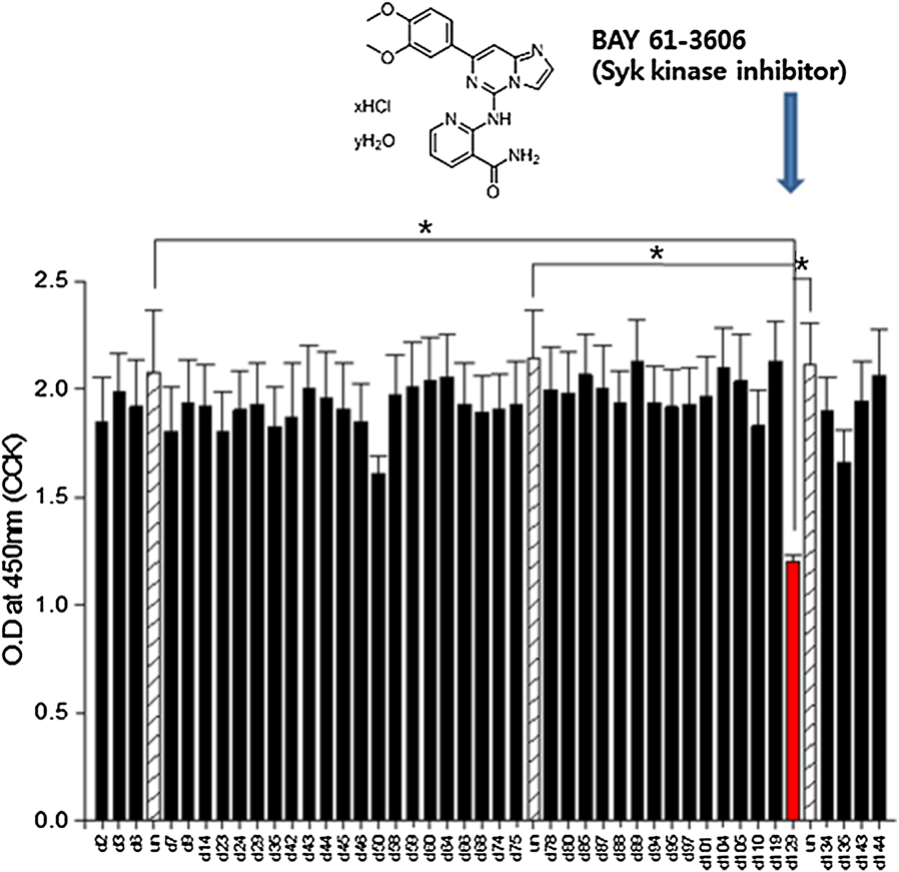
Initial screening of small molecules for VSMC proliferation suppression. A total of 43 small molecule inhibitors of various kinases with no reported anti-proliferative effect on VSMC were screened. Rat VSMCs were treated with 10 μM of each small molecule for 24 h, and the cellular proliferation was evaluated by cell counting kits (CCK-8). Un (vehicle): 10% FBS supplemented DMSO containing DMSO 2%, v/v, indicated as *white box* with oblique pattern. *Blue arrow* indicates the most effective small molecule. The quantitative data were expressed as the mean ± SEM of at least three independent experiments. Full names of each small molecule are listed in Table 1

For initial screening, through a literature search, 43 small molecules (Table 1), whose effects on VSMC proliferation and migration had not been reported, were selected from an in-house small molecule library mainly composed of commercially available inhibitors of six kinase subfamilies [10]. The effect of those 43 small molecules on VSMC proliferation was then examined. VSMCs were treated with each small molecule (10 μM) for 24 h, and cellular proliferation was determined by using a cell proliferation assay kit (CCK-8). The results indicated that the small molecule number 129 most significantly inhibited the proliferation of VSMCs, compared to the vehicle groups (DMSO 2%, v/v) (Fig. 1). The identity of compound 129 was BAY 61-3606 hydrochloride, a Syk inhibitor [11].

**Table 1:** List of small molecules used for initial screening

D2	*p*-Aminoclonidine hydrochloride
D3	Isoetharine mesylate salt
D6	N6-2-(4-aminophenyl) ethyladenosine
D7	ABT-702 dihydrochloride
D9	9-Cyclopentyladenine monomethansulfonate
D14	(−)-*p*-Bromolevamisole oxalate
D23	TBBz
D24	A3 hydrochloride
D29	Kenpaullone
D35	TG003
D42	DMNB (4,5-dimethoxy-2-nitrobenzaldehyde)
D43	R(+)-6-Bromo-APB hydrobromide
D44	S-(−)-Eticlopride hydrochloride
D45	BP897
D46	GW2974
D50	SCH-202676 hydrobromide
D58	I-OMe-Tyrphostin AG 538
D59	IRAK-1/4 inhibitor-1
D60	ZM 39923 hydrochloride
D64	Pinacidil monohydrate
D66	NS 8593 hydrochloride
D68	PD 98059
D74	CGP 57380
D75	Triamterene
D78	Amiloride hydrochloride hydrate
D80	Prilocaine hydrochloride
D85	*N*-*p*-Tosyl-l-phenylalanine chloromethyl ketone
D87	*N*-(3,3-Diphenylprophyl)glycinamide
D88	JX401
D89	SD-169
D94	Enoximone, >99% HPLC
D95	4-(3-Butoxy-4-methoxybenzyl)imidazolidin-2-one
D97	YM 976
D101	AS604850
D104	7,8-Dihydroxycoumarin
D105	8-(4-chlorophenylthio) adenosine 3ʹ,5ʹ-cyclic monophosphate sodium salt
D110	Hispidin
D119	BTO-1
D129	BAY 61-3606 hydrochloride
D134	Tyrphostin AG879
D135	SU4321
D143	Tetracaine hydrochloride
D144	Vanillic acid diethylamide

### BAY61 inhibits VSMC proliferation in a concentration and time-dependent manner

**Fig. 2:**
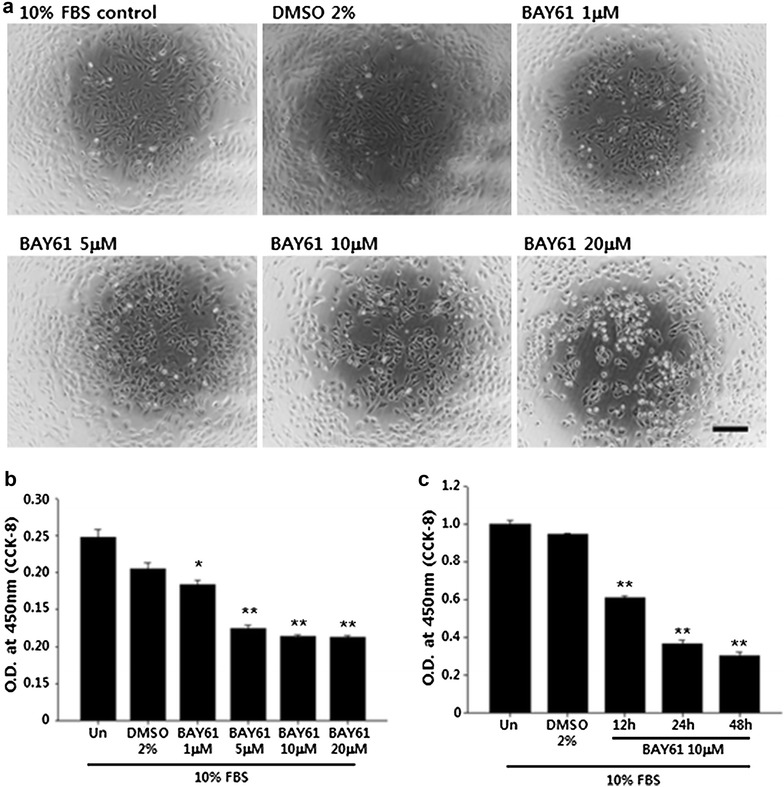
BAY61 inhibits VSMC proliferation in a concentration and time-dependent manner. **a** Representative images of VSMCs cultured with increasing concentrations of BAY61 for 24 h. *Scale bar* 200 μm. **b** The concentration-dependent effect of BAY61 on VSMC proliferation was determined after 24 h of BAY61 treatment as indicated using cell counting kit (CCK-8). *p < 0.05, **p < 0.01 compared to 10% FBS control. **c** To examine the time-dependent effect of BAY61, VSMCs were cultured in 10% FBS supplemented DMEM containing either vehicle (DMSO 2%, v/v) or 10 μM of BAY61 for up to 48 h. Cellular proliferation was determined by using CCK-8. *p < 0.05, **p < 0.01 compared to 10% FBS control. The quantitative data were expressed as the mean ± SEM of at least three independent experiments

To examine the concentration-dependent effect of the Syk inhibitor (BAY61), VSMCs were treated with increasing concentrations of BAY61 (1, 5, 10, and 20 μM) for 24 h. The cellular proliferation results indicated that BAY61 had a significant anti-proliferative effect at all concentrations tested, although the effect was more profound with concentrations of 5 μM or higher (Fig. 2a, b). At the concentration of 10 μM, BAY61 showed a significant anti-proliferative effect without any prominent morphological changes. On the other hand, at a concentration of 20 μM, the cells started to detach from the culture plate and to clump together. Therefore, for further experiments, 10 μM was used to examine the anti-proliferative effects of BAY61. To further examine the time-dependent effect of BAY61, VSMCs were treated with 10 μM of BAY61 for up to 48 h, and the cellular proliferation was decreased by BAY61 in a time-dependent manner (Fig. 2c).

### Anti-migratory effect of the Syk inhibitor

**Fig. 3:**
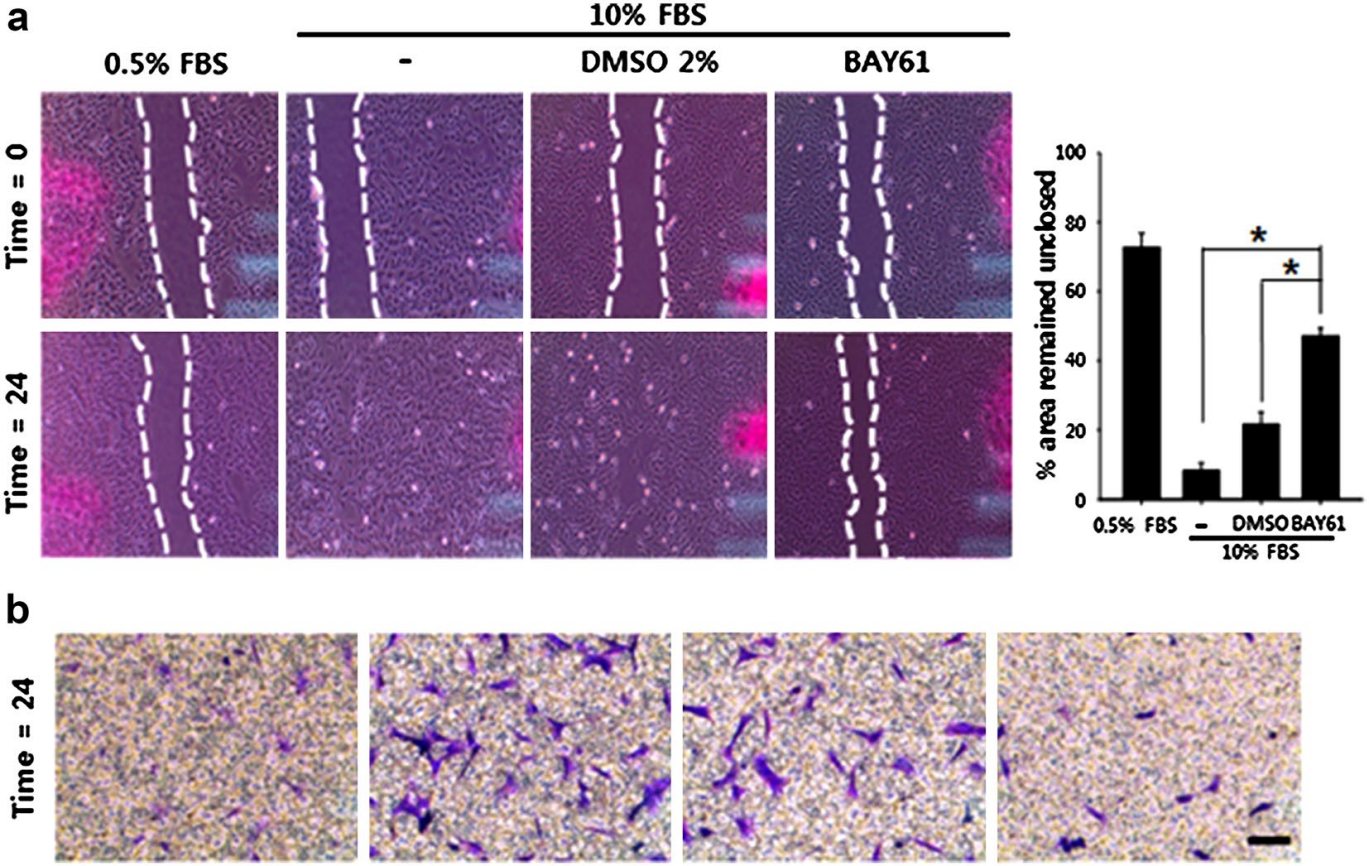
BAY61 suppresses migration of VSMCs. **a** Representative images of VSMC wound healing with or without BAY61 treatment. The cells were treated with either vehicle (DMSO 2%) or BAY61 (10 μM) after wound was generated by scratching with a yellow pipette tip. The cellular migration was monitored for 24 h. *Dotted line* indicates leading edge. *Scale bar* 200 μm. *p < 0.05. The quantitative data were expressed as the mean ± SEM of at least three independent experiments. **b** The anti-migratory effect of BAY61 (10 μM, 24 h) was evaluated by using transwell migration assay. *Scale bar* 200 μm

To evaluate the anti-migratory effect of BAY61, wound healing assays and transwell migration assays were conducted. According to the data, when the cells were stimulated with 10% FBS, the scratching wound produced with a yellow pipet tip was approximately 90% closed at 24 h, but the wound treated with BAY61 (10 μM) remained about 50% unclosed (Fig. 3a). Furthermore, BAY61 apparently decreased the number of cells that migrated through the transwell even in the presence of 10% serum (Fig. 3b).

### BAY61 suppresses VSMC sprouting from aortic rings ex vivo

**Fig. 4:**
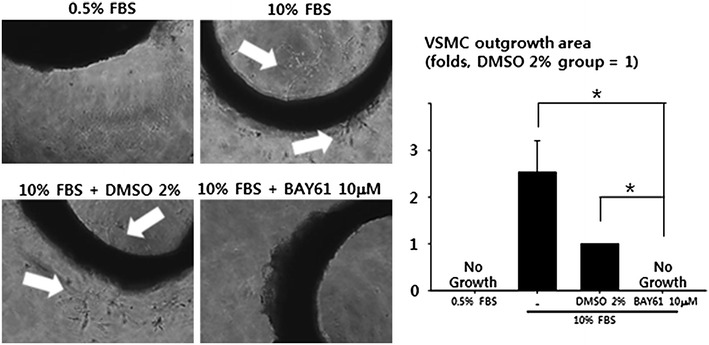
BAY61 attenuates VSMC outgrowth from endothelium denuded aortic ring. To examine the effect of BAY61 on the outgrowth of VSMCs from blood vessel, segments of endothelium-denuded aortic rings embedded in Matrigel were treated with either vehicle (DMSO 2%, v/v) or BAY61 (10 μM) for 7 days. On day 7, the pictures of VSMC sprouting from the aortic ring were taken. *White arrows* indicate the sprouting of VSMCs from the aortic rings. *p < 0.05. The quantitative data were expressed as the mean ± SEM of at least three independent experiments

To further investigate the effect of BAY61 on VSMC proliferation, an ex vivo aortic ring assay was performed. The endothelium-denuded thoracic aortas of rats were cut to prepare aortic rings with thickness of 1 mm. Matrigel-embedded aortic rings were cultured in 10% FBS-supplemented DMEM with either vehicle (DMSO 2%, v/v) or BAY61 (10 μM) for 7 days. VSMC sprouting from the endothelium-denuded aortic rings was inhibited by BAY61 even in the presence of 10% FBS in the culture medium (Fig. 4).

## Discussion

In the present study, through a screening of small molecule inhibitors of kinase subfamilies, we identified a Syk inhibitor as a potent anti-proliferative and migratory small molecule for VSMCs. Protein kinases are known to play critical roles in signaling pathways implicated in development, differentiation, death and proliferation of cells [12]. Therefore, it is reasonable to hypothesize that a certain kinase inhibitor can suppress proliferation and migration of VSMCs. Regarding the effect of BAY61 on cellular proliferation and migration, it has been reported that BAY61 significantly reduced the proliferation and migration of multiple myeloma cells [13] and it prevented airway epithelial cell proliferation and migration [14]. However, to our best knowledge, no previous study had reported anti-proliferative and migratory effects of this particular small molecule on VSMCs. Therefore, the present study is the first study to examine the effect of BAY61 on VSMC proliferation and migration.

Syk, along with zeta-chain associated kinase of 70 kDa (Zap-70), is a member of the Syk family of kinases that are activated downstream of Src kinases [15]. Syk is known to be mainly expressed in hematopoietic cells, but its expression has been also reported in epithelial cells and endothelial cells [16, 17]. The reported roles of Syk include, but not limited to, relaying adaptive immune receptor signaling, cellular adhesion, innate immune recognition, platelet activation, and vascular development [18]. Pertaining to the role of Syk in VSMC proliferation, it has been reported that inhibition or knockdown of Syk significantly prevented platelet-derived growth factor (PDGF)-induced proliferation and migration [19].

Our data also clearly demonstrated that the Syk inhibitor BAY61 significantly suppressed VSMC proliferation and migration. Previous study had reported that suppression of Syk activation led to subsequent inhibition of extracellular signal-regulated kinas (ERK) 1/2 signaling and p38 mitogen-activated protein kinase signaling [20]. Therefore, it is highly possible that BAY61 also inhibited VSMC proliferation and migration via suppression of ERK and p38 pathways. Although we did not examined detailed mechanisms of BAY61 and it is a major limitation of the present study, we are currently conducting a further study evaluating the in vivo efficacy of BAY61 in comparison to other well-known SMC proliferation inhibitor such as paclitaxel and the underlying mechanism of BAY61 will be also examined in detail.

Our ex vivo aortic ring assay result showed that BAY61 prevented sprouting of VSMCs, and furthermore, suppression of Syk has been suggested to be an effective way to control atherosclerosis via subsequent suppression of endothelin-1 production from endothelial cells [21]. Therefore, BAY61 is a strong candidate for an effective therapeutic agent targeting occlusive vascular diseases, such as restenosis, and it deserves further investigation to prove its in vivo efficacy and safety.

## Conclusions

In summary, as an effort to identify anti-proliferative agent to control occlusive vascular disease, this paper describes the anti-proliferative and migratory effects of BAY61, a Syk inhibitor, on VSMCs for the first time. The selected Syk inhibitor significantly suppressed VSMC proliferation and migration in vitro and ex vivo. These data suggest that the BAY61 may be a potent therapeutic agent for the treatment of occlusive vascular disease and warrants further studies to evaluate its in vivo efficacy and detailed underlying mechanism.

## Methods

### Isolation and culture of rat aortic VSMCs

All experimental procedures for animal studies were approved by the Committee for the Care and Use of Laboratory Animals, Yonsei University College of Medicine, and were performed in accordance with the Committee’s guidelines and regulations for animal care. Rat aortic VSMCs were isolated and cultured using methods previously published [22]. The thoracic aortas from 6- to 8-week-old Sprague–Dawley rats were removed and transferred to serum-free Dulbecco’s modified Eagle’s medium (DMEM; Invitrogen, USA) containing 100 units/ml penicillin and 100 mg/ml streptomycin. The connective tissues were removed, and the aorta was transferred to a petri dish containing 5 ml of an enzyme dissociation mixture containing DMEM with 1 mg/ml collagenase type I (Sigma, USA) and 0.5 mg/ml elastase (USB Bioscience, USA) and was incubated for 30 min at 37 °C. The adventitia was stripped with forceps under a microscope. The aorta was transferred to a plastic tube containing 5 ml enzyme dissociation mixture and incubated for 2 h at 37 °C. The suspension was centrifuged (1500 rpm for 10 min), and the pellet was re-suspended with 10% fetal bovine serum (FBS) containing DMEM with. Rat aortic SMCs were cultured in DMEM supplemented with 10% FBS, 100 IU/ml penicillin and 100 mg/ml streptomycin in 75 cm^2^ flasks at 37 °C in a humidified atmosphere of 95% air and 5% CO_2_ (Forma Scientific, USA).

### Primary screening of small molecules

From an in-house small molecule library mainly composed of inhibitors of six major kinase subfamilies, 43 candidate small molecules with no known anti-proliferative effects on VSMCs were selected based on a published literature search using Pubmed (http://www.ncbi.nlm.nih.gov/pubmed). For primary screening, VSMCs were seeded in 48-well culture plates using DMEM with 10% FBS and then treated with 10 μM of each candidate compound for 24 h. The anti-proliferative effects of the candidate compounds were determined by a cell proliferation assay.

### Cell proliferation assay (cell counting kit 8, CCK 8)

To determine cell proliferation, cell proliferation assay using a WST-8 [2-(2-methoxy-4-nitrophenyl)-3-(4-nitrophenyl)-5-(2,4-disulfophenyl)-*2H*-tetrazolium, monosodium salt] solution (CCK-8, Dojindo, Japan) was conducted following protocol previously published [23]. Briefly, WST-8 solution was added to each well [10% (v/v)] and incubated at 37 °C for 2 h to allow for the formation of WST-8 formazan. The absorbance of a water soluble formazan dye was measured at 450 nm using a microplate reader (Molecular Devices, USA).

### Wound healing assay

Wound healing assay, also as known as in vitro scratch assay, was conducted according to the protocol previously published [24]. VSMCs were plated at a density of 8 × 10^4^ cells/well in six-well plates. After the cells had reached 90% confluence, wounds were produced by scratching with 200 μl pipette tips. The leading edge of the wounds was marked as a baseline. The medium was replaced with or without the chemical compound, and the cells were incubated for up to 48 h. Images were captured using an Axiovert 40C inverted microscope (Carl Zeiss, Germany) equipped with a Powershot A640 digital camera (Canon, Japan). The percent area remained unclosed (area between the leading edges at a given time point/area between the leading edges at baseline × 100) was measured.

### Transwell migration assay

Transwell migration assay was performed according to the protocol previously published [25]. VSMCs (8 × 10^3^ cells) were seeded onto the upper chamber of a transwell filter with 8 μm pores (Costar Corning, USA). The cells were deprived of serum for 24 h, and a candidate chemical compound was added to the lower chamber. Transwell chambers were incubated at 37 °C for 24 h. After incubation, the cells that migrated through the pores of the filter were stained with 0.25% crystal violet. Non-migrating cells on the upper side of the filter were removed with cotton swabs.

### Western Blot analysis

VSMCs cultured in 60-mm dishes were treated with or without the selected chemical compound. Proteins were separated in a 10% SDS–polyacrylamide gel and transferred to PVDF membranes (Millipore, USA). After blocking the membrane with TBS-T (TBS-tween 20, 0.1% tween 20) containing 5% (w/v) non-fat dried skimmed milk powder for 1 h at room temperature, the membranes were washed twice with TBS-T and incubated with primary antibodies for 1 h at room temperature or overnight at 4 °C. The membranes were washed three times with TBS-T for 5 min and incubated with horseradish peroxidase (HRP)-conjugated secondary antibody for 1 h at room temperature. After extensive washing, the bands were detected using enhanced chemiluminescence (ECL®) reagent (AbClon, Republic of Korea). The band intensities were detected using a Photo-Image System (Molecular Dynamics, Canada). The primary antibodies were from Cell Signaling (cyclin D1:2978, p-ERK:9101, ERK:9102, p-Akt:9271, Akt:9272), Santa Cruz [PCNA (proliferating cell nuclear antigen):sc-56, p-Rb:sc-16670-R, Rb:sc-50], and Abcam (β-actin:ab8227).

### Ex vivo aortic ring assay

Aortic ring assay to monitor the outgrowth of VSMCs was conducted following a published protocol with modifications [26]. Briefly, the thoracic aortas from 6- to 8-week-old Sprague–Dawley rats were removed and transferred to serum-free DMEM. The lining endothelium was denuded using a 2-Fr Fogarty balloon catheter (Baxter, USA) to minimize the possibility of endothelial cell sprouting during the ring assay. The aortas were washed by gradually passing through PBS three times. After removing perivascular adipose tissue, each aorta was cut into segments of aortic rings 1 mm in length that were placed in Matrigel (BD Biosciences, USA). The aortic rings were incubated with or without the selected chemical compound. Over the next 7 days, the aorta rings were monitored daily for the sprouting of VSMCs using a microscope.

### Statistical analysis

Quantitative data were expressed as the mean ± SEM (standard error of measurement). For statistical analysis, one-way ANOVA with Bonferroni correction was performed using OriginPro 8 SR4 software (ver. 8.0951, OriginLab Corporation, USA) if there were more than three groups. A *p* value of less than 0.05 was considered statistically significant.
